# Serum levels of markers in early detection of prostate cancer (pilote study)

**DOI:** 10.1186/1878-5085-5-S1-A37

**Published:** 2014-02-11

**Authors:** Radka Fuchsova, Ondrej Topolcan, Jindra Vrzalova, Milan Hora, Radek Kucera, Olga Dolejsova, Ondrej Hes, Jiri Ferda

**Affiliations:** 1 Immunoanalytical Laboratory, Department of Nuclear Medicine, University Hospital in Pilsen, Czech Republic; 2 Department of Urology, Faculty of Medicine and University Hospital in Pilsen, Charles University in Prague, Czech Republic; 3 Department of Pathology, Faculty of Medicine and University Hospital in Pilsen, Charles University in Prague, Czech Republic; 4 Department of Radiology, Faculty of Medicine and University Hospital in Pilsen, Charles University in Prague, Czech Republic

## Scientific objectives

Monitoring changes in the levels of biomarkers PSA, %freePSA, [-2] proPSA and calculation of PHI in the diagnostic algorithm of early prostate cancer.

## Technological approaches

The Immunoanalytical Laboratory of University Hospital in Pilsen examined sera of 76 patients from the Urology department of the University Hospital with suspected prostate cancer who have undergone TRUS biopsy. We assessed the levels of PSA and, if the interval of PSA was between 0-30 ng/mL, we also assessed the levels of freePSA, [-2]proPSA and we calculated %freePSA and Prostate Health Index (PHI). The monitored biomarkers were measured using the chemiluminescent DxI 800 instrument (Beckman Coulter, USA). The peripheral blood was drawn by VACUETTE ® (Greiner Bio-One, Austria). All specimens were immediately aliquoted, frozen and stored at -80°C. Samples were thaw only ones just before the processing. SAS 9.2 software was used for all statistical analysis.

## Results interpretation

**Figure 1:**
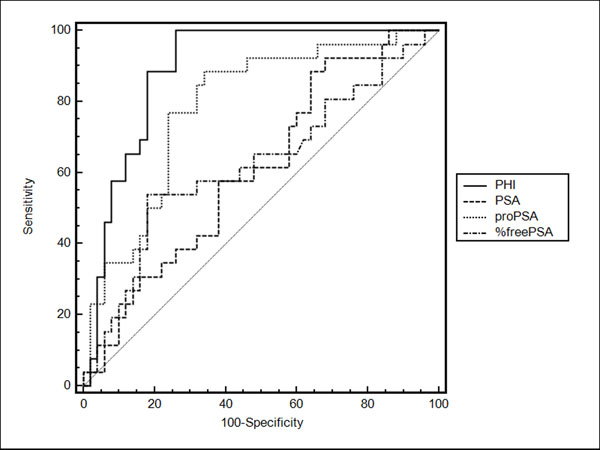
ROC curves of prostatic parameters

We found statistically significant increased levels of [-2]proPSA and PHI in patients diagnosed with prostate cancer by prostate biopsy vs. patients with benign prostate hypertrophy ([-2]proPSA median 14 vs. 27 pg/mL, PHI median 35 vs. 77). In the contrary, we did not find any significant difference in tPSA and %freePSA (median tPSA 7.1 vs. 7.7 ng/mL and %freePSA 16 vs.11.4%). According to receiver operating characteristic (ROC) curves is PHI the best parameter for distinguishing benign and malignant prostate tumor (Figure 1).

## Outlook and Expert recommendations

The assessment of [-2]proPSA and the calculation of PHI appear to be of great benefit for a more accurate differential diagnosis of benign hyperplasia. PHI calculation leads to a biopsy reduction and it is expected that the verification in a larger file of patients will have significant impact for the assessment of tumor aggressiveness.

